# Supplementary material for: Analysis of multi-level barriers to physical activity among nursing students using regularized regression
Source: PLoS One. 2024 May 24;19(5):e0304214. doi: 10.1371/journal.pone.0304214 (PMC11125535; doi:10.1371/journal.pone.0304214)
Supplement: S1 File — (PDF) [file pone.0304214.s001.pdf]

The questions below are to capture information about your level of education.

What is your year of study?	◯ Freshman<br>◯ Sophomore<br>◯ Junior<br>◯ Senior<br>◯ Other
Please specify.	______________________________
Are you a UMass accelerated nursing program student?	◯ Yes<br>◯ No

To answer any missed questions or to check your answers, please hit "Previous Page". To proceed with the survey, please hit "Next Page". To continue the survey later, please hit "Save and Return Later".

Section 1: Individual Physical Activity Questionnaire

This section includes questions about physical activities (PA) you do in your everyday life. There are 5 parts (sets of questions):

Part 1: job-related PA
Part 2: transportation PA
Part 3: housework and care giving PA
Part 4: recreation and leisure-time PA
Part 5: sitting

The questions will ask about vigorous and moderate PA that you did in the last 7 days.

Vigorous = activities that take hard physical effort and make you breathe much harder than normal.
Moderate = activities that take moderate physical effort and make you breathe somewhat harder than normal.

PART 1: JOB-RELATED PHYSICAL ACTIVITY

Part 1 is about your work. Work can refer to any paid or unpaid job that you consider your main occupation. At the end of this section, you will have the opportunity to describe how you define work.

Do you currently have a paid or unpaid job that you consider your main occupation?

○ Yes
○ No

The next questions are about all the physical activity you did in the last 7 days as part of your paid or unpaid work. This does not include traveling to and from work.

During the last 7 days, on how many days did you do vigorous physical activities like heavy lifting, digging, heavy construction, or climbing up stairs as part of your work? Think about only those physical activities that you did for at least 10 minutes at a time.

____

How much time did you usually spend on one of those days doing vigorous physical activities as part of your work? Please enter time in minutes.

Example: 1 hr 30 mins = 90 mins

____

Again, think about only those physical activities that you did for at least 10 minutes at a time. During the last 7 days, on how many days did you do moderate physical activities like carrying light loads as part of your work? Please do not include walking.

____

How much time did you usually spend on one of those days doing moderate physical activities as part of your work? Please enter time in minutes.	________________________
During the last 7 days, on how many days did you walk for at least 10 minutes at a time as part of your work? Please do not count any walking you did to travel to or from work.	________________________
How much time did you usually spend on one of those days walking as part of your work? Please enter time in minutes.	________________________

To answer any missed questions or to check your answers, please hit "Previous Page". To proceed with the survey, please hit "Next Page". To continue the survey later, please hit "Save and Return Later".

PART 2: TRANSPORTATION PHYSICAL ACTIVITY

Part 2 includes questions about how you traveled from place to place, including to places like work,
stores, movies, and so on.

During the last 7 days, on how many days did you travel in a motor vehicle like a train, bus, car, or tram? __________________________________

How much time did you usually spend on one of those days traveling in a train, bus, car, tram, or other kind of motor vehicle? Please enter time in minutes. __________________________________

Now think only about the bicycling and walking you might have done to travel to and from work, to do errands, or to go from place to place.

During the last 7 days, on how many days did you bicycle for at least 10 minutes at a time to go from place to place? __________________________________

How much time did you usually spend on one of those days to bicycle from place to place? Please enter time in minutes. __________________________________

During the last 7 days, on how many days did you walk for at least 10 minutes at a time to go from place to place? __________________________________

How much time did you usually spend on one of those days walking from place to place? Please enter time in minutes. __________________________________

To answer any missed questions or to check your answers, please hit "Previous Page". To proceed with the survey, please hit "Next Page". To continue the survey later, please hit "Save and Return Later".

PART 3: HOUSEWORK, HOUSE MAINTENANCE, AND CARING FOR FAMILY

Part 3 is about some of the physical activities you might have done in the last 7 days in and around your home, like housework, gardening, yard work, general maintenance work, and caring for your family.

Think about only those physical activities that you did for at least 10 minutes at a time. During the last 7 days, on how many days did you do vigorous physical activities like heavy lifting, chopping wood, shoveling snow, or digging in the garden or yard?	__________________________________
How much time did you usually spend on one of those days doing vigorous physical activities in the garden or yard? Please enter time in minutes.	__________________________________
Again, think about only those physical activities that you did for at least 10 minutes at a time. During the last 7 days, on how many days did you do moderate activities like carrying light loads, sweeping, washing windows, and raking in the garden or yard?	__________________________________
How much time did you usually spend on one of those days doing moderate physical activities in the garden or yard? Please enter time in minutes.	__________________________________
Once again, think about only those physical activities that you did for at least 10 minutes at a time. During the last 7 days, on how many days did you do moderate activities like carrying light loads, washing windows, scrubbing floors and sweeping inside your home?	__________________________________
How much time did you usually spend on one of those days doing moderate physical activities inside your home? Please enter time in minutes.	__________________________________

To answer any missed questions or to check your answers, please hit "Previous Page". To proceed with the survey, please hit "Next Page". To continue the survey later, please hit "Save and Return Later".

PART 4: RECREATION, SPORT, AND LEISURE-TIME PHYSICAL ACTIVITY

Part 4 is about all the physical activities that you did in the last 7 days solely for recreation, sport, exercise or leisure. Please do not include any activities you have already mentioned.

Not counting any walking you have already mentioned, during the last 7 days, on how many days did you walk for at least 10 minutes at a time in your leisure time?	______________________
How much time did you usually spend on one of those days walking in your leisure time? Please enter time in minutes.	______________________
Think about only those physical activities that you did for at least 10 minutes at a time. During the last 7 days, on how many days did you do vigorous physical activities like aerobics, running, fast bicycling, or fast swimming in your leisure time?	______________________
How much time did you usually spend on one of those days doing vigorous physical activities in your leisure time? Please enter time in minutes.	______________________
Again, think about only those physical activities that you did for at least 10 minutes at a time. During the last 7 days, on how many days did you do moderate physical activities like bicycling at a regular pace, swimming at a regular pace, and doubles tennis in your leisure time?	______________________
How much time did you usually spend on one of those days doing moderate physical activities in your leisure time? Please enter time in minutes.	______________________

To answer any missed questions or to check your answers, please hit "Previous Page". To proceed with the survey, please hit "Next Page". To continue the survey later, please hit "Save and Return Later".

PART 5: TIME SPENT SITTING

Part 5 is about the time you spend sitting while at work, at home, while doing course work and during leisure time. This may include time spent sitting at a chair, couch and lying down. Do not include any time spent sitting in a motor vehicle (e.g., sitting during transportation).

During the last 7 days, how much time did you usually spend sitting on a weekday (eg Monday, Tuesday...Friday)? Please enter time in minutes. __________________________________

During the last 7 days, how much time did you usually spend sitting on a weekend day (Saturday or Sunday)? Please enter time in minutes. __________________________________

What is your primary occupation that you define as "work" when answered questions involving activities during work in this Section? Select one option.

- ○ Being a student
- ○ Part-time work (other than being a student)
- ○ Full-time work (other than being a student)
- ○ Other

Please specify. __________________________________

To answer any missed questions or to check your answers, please hit "Previous Page". To proceed with the survey, please hit "Next Page". To continue the survey later, please hit "Save and Return Later".

Section 2: Motives for Physical Activity

In this section, we are interested in knowing why you do physical activity.

I do physical activity:

	1. not true at all	2.	3.	4. somewhat true	5.	6.	7. very true
Because I want to be physically fit	○	○	○	○	○	○	○
Because it's fun	○	○	○	○	○	○	○
Because I like engaging in activities which physically challenge me	○	○	○	○	○	○	○
Because I want to obtain new skills	○	○	○	○	○	○	○
Because I want to look or maintain weight so I look better	○	○	○	○	○	○	○
Because I want to be with my friends	○	○	○	○	○	○	○
Because I like to do this activity	○	○	○	○	○	○	○
Because I want to improve existing skills	○	○	○	○	○	○	○
Because I like the challenge	○	○	○	○	○	○	○
Because I want to define my muscles so I look better	○	○	○	○	○	○	○
Because it makes me happy	○	○	○	○	○	○	○
Because I want to keep up my current skill level	○	○	○	○	○	○	○
Because I want to have more energy	○	○	○	○	○	○	○
Because I like activities which are physically challenging	○	○	○	○	○	○	○
Because I like to be with others who are interested in this activity	○	○	○	○	○	○	○
Because I want to improve my cardiovascular fitness	○	○	○	○	○	○	○
Because I want to improve my appearance	○	○	○	○	○	○	○
Because I think it's interesting	○	○	○	○	○	○	○
Because I want to maintain my physical strength to live a healthy life	○	○	○	○	○	○	○

Because I want to be attractive to others	○	○	○	○	○	○	○
Because I want to meet new people	○	○	○	○	○	○	○
Because I enjoy this activity	○	○	○	○	○	○	○
Because I want to maintain my physical health and well-being	○	○	○	○	○	○	○
Because I want to improve my body shape	○	○	○	○	○	○	○
Because I want to get better at my activity	○	○	○	○	○	○	○
Because I find this activity stimulating	○	○	○	○	○	○	○
Because I will feel physically unattractive if I don't	○	○	○	○	○	○	○
Because my friends want me to	○	○	○	○	○	○	○
Because I like the excitement of participation	○	○	○	○	○	○	○
Because I enjoy spending time with others doing this activity	○	○	○	○	○	○	○

To answer any missed questions or to check your answers, please hit "Previous Page". To proceed with the survey, please hit "Next Page". To continue the survey later, please hit "Save and Return Later".

Section 3

Below are statements that relate to ideas about exercise. Please indicate the degree to which you agree or disagree with the statements.

	Strongly Disagree	Disagree	Agree	Strongly Agree
Exercising takes too much of my time	◯	◯	◯	◯
Exercise tires me	◯	◯	◯	◯
Places for me to exercise are too far away	◯	◯	◯	◯
I am too embarrassed to exercise	◯	◯	◯	◯
It costs too much to exercise	◯	◯	◯	◯
Exercise facilities do not have convenient schedules for me	◯	◯	◯	◯
I am fatigued by exercise	◯	◯	◯	◯
My spouse (or significant other) does not encourage exercising	◯	◯	◯	◯
Exercise takes too much times from family relationships	◯	◯	◯	◯
I think people in exercise clothes look funny	◯	◯	◯	◯
My family members do not encourage me to exercise	◯	◯	◯	◯
Exercise takes too much time from my family responsibilities	◯	◯	◯	◯
Exercise is hard work for me	◯	◯	◯	◯
There are too few places for me to exercise	◯	◯	◯	◯

To answer any missed questions or to check your answers, please hit "Previous Page". To proceed with the survey, please hit "Next Page". To continue the survey later, please hit "Save and Return Later".

Section 4: Physical Activity and Social Support

Please indicate the degree to which following statements are true for you.

	Never true	Rarely true	Sometimes but infrequently true	Neutral	Sometimes true	Usually true	Always true
I have someone who can provide reassurance in the activity/activities	○	○	○	○	○	○	○
There is someone that provides me with positive feedback in the activity/activities	○	○	○	○	○	○	○
There is someone who understands my problems/worries about the activity/activities	○	○	○	○	○	○	○
I have someone with whom I can relate to in the activity/activities	○	○	○	○	○	○	○
I set expectations based on the performance of others in the activity/activities	○	○	○	○	○	○	○
I want to know competition results (i.e., race results), times, duration, weights, or actions of others in the activity/activities	○	○	○	○	○	○	○
I compare myself to others in the activity/activities	○	○	○	○	○	○	○
I use social media to find other people's performance in the activity/activities to compare to my own	○	○	○	○	○	○	○
I read articles about the activity/activities	○	○	○	○	○	○	○
I seek out information from others to get better at the activity/activities	○	○	○	○	○	○	○
I talk to people for assistance or to improve technique in the activity/activities	○	○	○	○	○	○	○
I attend clinics, classes, and workshops to learn about the activity/activities	○	○	○	○	○	○	○

I am a part of a core group of people who do the activity/activities	○	○	○	○	○	○	○
When not engaging in the activity/activities, I still spend time with people that I met while in the activity/activities	○	○	○	○	○	○	○
I feel a sense of belonging to a group that also does the activity/activities I do	○	○	○	○	○	○	○
I can find someone to do the activity/activities with, even outside of my friends	○	○	○	○	○	○	○
I can get help traveling if needed to perform the activity/activities	○	○	○	○	○	○	○
I have someone that could loan or give me something to help carry out the activity/activities I do	○	○	○	○	○	○	○
I have someone who would watch my child(ren) or pets if needed for me to engage in the activity/activities	○	○	○	○	○	○	○
I can find someone to help on short notice so that I can engage in the activity/activities	○	○	○	○	○	○	○

To answer any missed questions or to check your answers, please hit "Previous Page". To proceed with the survey, please hit "Next Page". To continue the survey later, please hit "Save and Return Later".

Section 5: Health Data

"Health data" refers to information about an individual's health and wellness (eg: physical activity, sleep, movement, stress etc). Health data can be collected through a device, including smartphones and wearables, and can be shared with other individuals.

We would like to know about your opinion on how health data should be collected and shared.

Would you be willing to share your health data from a wearable device with your health care provider?	◯ Yes<br>◯ No
Would you be willing to share your health data from a wearable device with your family?	◯ Yes<br>◯ No
Would you be willing to share your health data from a wearable device with your friends?	◯ Yes<br>◯ No
In the past month, how often have you used a wearable device that captures health data?	◯ Every day<br>◯ Almost every day<br>◯ 1-2 times per week<br>◯ < 1 time per week<br>◯ Not in the past month<br>◯ Do not own a wearable device that captures health data

To answer any missed questions or to check your answers, please hit "Previous Page". To proceed with the survey, please hit "Next Page". To continue the survey later, please hit "Save and Return Later".

Section 6: Internet and Device Usage

We are interested in knowing about your internet and device usage as related to collecting and sharing health data. Please answer the following sets of questions. Hit "Next Page" to proceed.

Has your tablet or smartphone...

	Yes	No
Helped you track progress on a health-related goal, such as quitting smoking, losing weight, or increasing physical activity?	○	○
Helped you make a decision about how to treat an illness or condition?	○	○
Helped you in discussions with your health care provider?	○	○

Other than a tablet or smartphone, have you used an wearable device to monitor or track your health within the last 12 months? Examples include Fitbit, blood glucose meters, and blood pressure monitors.

- ○ Yes
- ○ No

To answer any missed questions or to check your answers, please hit "Previous Page". To proceed with the survey, please hit "Next Page". To continue the survey later, please hit "Save and Return Later".

Has your wearable device...

	Yes	No
Helped you track progress on a health-related goal, such as quitting smoking, losing weight, or increasing physical activity?	◯	◯
Helped you make a decision about how to treat an illness or condition?	◯	◯
Helped you in discussions with your health care provider?	◯	◯

Have you sent a text message to or received a text message from a doctor or other health care professional within the last 12 months?

◯ Yes
◯ No
◯ Don't know

To answer any missed questions or to check your answers, please hit "Previous Page". To proceed with the survey, please hit "Next Page". To continue the survey later, please hit "Save and Return Later".

Section 7: General Health

We are interested in knowing about your general health and wellness. Please answer the following set of questions.

Please enter your height (inches).
(For example: 5 ft 6 in = 5 ft * 12 in + 6 in = 66 inches) ________________________________

Please enter your weight (pounds). ________________________________

To answer any missed questions or to check your answers, please hit "Previous Page". To proceed with the survey, please hit "Next Page". To continue the survey later, please hit "Save and Return Later".

Section 8: Common Health Problems

The following is a list of common problems. Please indicate if you currently have the problem in the first column. If you do not have the problem, skip to next problem.

If you do have the problem, please indicate in the second column if you receive medications or some other type of treatment for the problem.

In the third column indicate if the problem limits any of your activities.

Finally, indicate all medical conditions that are not listed under "other medical problems" at the end of the page.

Do you have the problem?

	Yes	No
Heart disease	○	○
High blood pressure	○	○
Lung disease	○	○
Diabetes	○	○
Ulcer or stomach disease	○	○
Kidney disease	○	○
Liver disease	○	○
Anemia or other blood disease	○	○
Cancer	○	○
Depression	○	○
Osteoarthritis, degenerative arthritis	○	○
Back pain	○	○
Rheumatoid arthritis	○	○
Other medical problems	○	○

Please write in: ______________________________

To answer any missed questions or to check your answers, please hit "Previous Page". To proceed with the survey, please hit "Next Page". To continue the survey later, please hit "Save and Return Later".

Do you receive treatment for it?

	Yes	No
Heart disease	◯	◯
High blood pressure	◯	◯
Lung disease	◯	◯
Diabetes	◯	◯
Ulcer or stomach disease	◯	◯
Kidney disease	◯	◯
Liver disease	◯	◯
Anemia or other blood disease	◯	◯
Cancer	◯	◯
Depression	◯	◯
Osteoarthritis, degenerative arthritis	◯	◯
Back Pain	◯	◯
Rheumatoid arthritis	◯	◯
Other medical problems	◯	◯

To answer any missed questions or to check your answers, please hit "Previous Page". To proceed with the survey, please hit "Next Page". To continue the survey later, please hit "Save and Return Later".

Does it limit your activities?

	Yes	No
Heart disease	◯	◯
High blood pressure	◯	◯
Lung disease	◯	◯
Diabetes	◯	◯
Ulcer or stomach disease	◯	◯
Kidney disease	◯	◯
Liver disease	◯	◯
Anemia or other blood disease	◯	◯
Cancer	◯	◯
Depression	◯	◯
Osteoarthritis, degenerative arthritis	◯	◯
Back pain	◯	◯
Rheumatoid arthritis	◯	◯
Other medical problems	◯	◯

To answer any missed questions or to check your answers, please hit "Previous Page". To proceed with the survey, please hit "Next Page". To continue the survey later, please hit "Save and Return Later".

Section 9: Sleep Quality

The following question refers to your overall sleep quality for the majority of nights in the past 7 days ONLY.

Please think about the quality of your sleep overall, such as how many hours of sleep you got, how easily you fell asleep, how often you woke up during the night (except to go to the bathroom), how often you woke up earlier than you had to in the morning, and how refreshing your sleep was.

Please rate this question on a scale of 0-10 (0 -- Terrible, 1-3 -- Poor, 4-6 -- Fair, 7-9 -- Good, 10 -- Excellent).

	0	1	2	3	4	5	6	7	8	9	10
During the past 7 days, how would you rate your overall sleep quality?	○	○	○	○	○	○	○	○	○	○	○

To answer any missed questions or to check your answers, please hit "Previous Page". To proceed with the survey, please hit "Next Page". To continue the survey later, please hit "Save and Return Later".

Section 10: State of Health

Would you say that in general your health is excellent, very good, good, fair or poor?	◯ Excellent<br>◯ Very good<br>◯ Good<br>◯ Fair<br>◯ Poor
Now thinking about your physical health, which includes physical illness and injury, how many days during the past 30 days was your physical health not good? Write 0 if none.	__________________________________
Now thinking about your mental health, which includes stress, depression, and problems with emotions, how many days during the past 30 days was your mental health not good? Write 0 if none.	__________________________________
During the past 30 days, approximately how many days did poor physical or mental health keep you from doing your usual activities, such as self-care, work, or recreation? Write 0 if none.	__________________________________

To answer any missed questions or to check your answers, please hit "Previous Page". To proceed with the survey, please hit "Next Page". To continue the survey later, please hit "Save and Return Later".

Section 11: Demographics

The following questions will ask about demographics, country of origin, language, and education.

What was your biological sex at birth?
- ◯ Female
- ◯ Male
- ◯ Intersex
- ◯ None of these describe me
- ◯ Prefer not to answer

What is your age?

____

What is your current zipcode?

____

What is your best estimate of the total income of all family members from all sources, before taxes, in the last calendar year (in US dollars $)?

____

Where were you born?
- ◯ In the United States
- ◯ Outside the United States

Print name of state.

____

Print U.S. Territory (e.g., Puerto Rico, U.S. Virgin Islands, Guam) or name of foreign country, etc.

____

What is your race?
- ◯ White
- ◯ Black or African American
- ◯ American Indian or Alaska Native
- ◯ Chinese
- ◯ Filipino
- ◯ Asian Indian
- ◯ Vietnamese
- ◯ Korean
- ◯ Japanese
- ◯ Other Asian
- ◯ Native Hawaiian
- ◯ Samoan
- ◯ Chamorro
- ◯ Other Pacific Islander
- ◯ Some other race

Please enter race of origin.

____

Are you of Hispanic, Latino or Spanish origin?
- ◯ No, not of Hispanic, Latino, or Spanish origin
- ◯ Yes, Mexican, Mexican American, Chicano
- ◯ Yes, Puerto Rican
- ◯ Yes, Cuban
- ◯ Yes, another Hispanic, Latino, or Spanish origin

Do you speak a language other than English at home?
- ◯ Yes
- ◯ No

What language do you speak at home?

____

Since you speak a language other than English at home, we are interested in your own opinion of how well you speak English. Would you say you speak English...?

- ◯ Very well
- ◯ Well
- ◯ Not well
- ◯ Not at all
- ◯ Don't know

What is your highest completed educational degree?

- ◯ High school diploma/GED
- ◯ Associates
- ◯ Bachelors
- ◯ Masters or other graduate degree

To answer any missed questions or to check your answers, please hit "Previous Page". To proceed with the survey, please hit "Next Page". To continue the survey later, please hit "Save and Return Later".

Please provide us with your name and email. This information is not a part of the study and will only be used for reimbursement purposes.

First name. ______________________________

Last name. ______________________________

Email. ______________________________

This is the end of the survey. When you are done completing the survey, please click the Submit button below. Thank you for your participation.
