# Supplementary material for: Analysis of multi-level barriers to physical activity among nursing students using regularized regression
Source: PLoS One. 2024 May 24;19(5):e0304214. doi: 10.1371/journal.pone.0304214 (PMC11125535; doi:10.1371/journal.pone.0304214)
Supplement: S1 Table — (PDF) [file pone.0304214.s002.pdf]

S1 Table: Missing Value Percentages for Independent Variables

Independent Variable	Description	Missing Values (%)
x_1	Interest in PA as a motive	0.18
x_2	Total leisure-time PA (MET-minutes/week)	22.29
x_3	Emotional support (e.g., advice, positive messages, encouragement)	0.00
x_4	Instrumental support (e.g., having appropriate gear, financial support)	0.61
x_5	Validation support (e.g., seeking out others for social comparison, relative status in a group)	0.00
x_6	Total family income in the last year	11.66
x_7, x_8	Highest educational degree attained	0.00
x_9	Makes health decisions using a smartphone or tablet	0.00
x_{10}	Tracks health with a wearable device	0.00
x_{11}	Makes health decisions with a wearable device	0.01
x_{12}, x_{13}	Texting a health provider	0.01

Missing values were present for independent variables, given by x_i for $i = 1, 2, ..., 13$. Variables x_1, x_2, x_3, x_4, and x_5 are composite variables formed through summation of constituent variables. Missing value percentages for these composite variables are calculated by finding the missing value percentage of all its constituent variables. Variables that are composed of multiple factor levels (x_7, x_8; x_{12}, x_{13}) are considered to represent one variable, and therefore have the same missing value percentage.
